# Supplementary material for: Small-scale layered structures at the inner core boundary
Source: Nat Commun. 2023 Oct 11;14:6362. doi: 10.1038/s41467-023-42177-7 (PMC10567691; doi:10.1038/s41467-023-42177-7)
Supplement: Supplementary file 1 — Supplementary Information [file 41467_2023_42177_MOESM1_ESM.pdf]

Supplementary Information for

Small-scale Layered Structures at the Inner Core Boundary

Baolong Zhang[1,2], Sidao Ni[1*], Wenbo Wu[3], Zhichao Shen[3], Wenzhong Wang[4], Daoyuan Sun[4], Zhongqing Wu[4]

e-mail: sdni@whigg.ac.cn

Supplementary Table 1: Event list used in this study.

Event ID	Origin Time(UTC)	Mw	Depth (km)	Lat(°)	Lon(°)	Trace Number
Event 1	2019-08-08,00:45:25.50	5.8	226	36.4905	70.1291	42
Event 2	2013-05-24,14:56:31.60	6.7	632	52.1357	151.5688	365
Event 3	2016-07-23,01:00:21.54	5.8	417	47.6801	146.9368	19
Event 4	2017-09-03,03:30:00.88	6.3	0.0	41.3199	129.0491	22

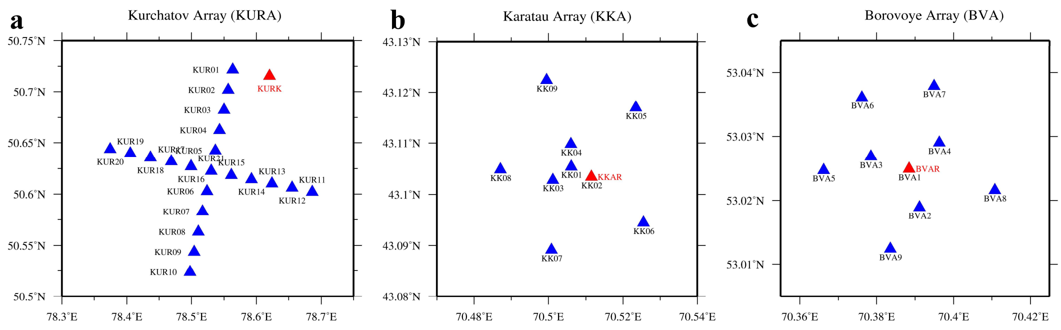

Supplementary Fig. 1. Zoomed in map of three small aperture dense arrays in Central Asia. The red triangles denote the three-component broadband stations in the dense arrays.

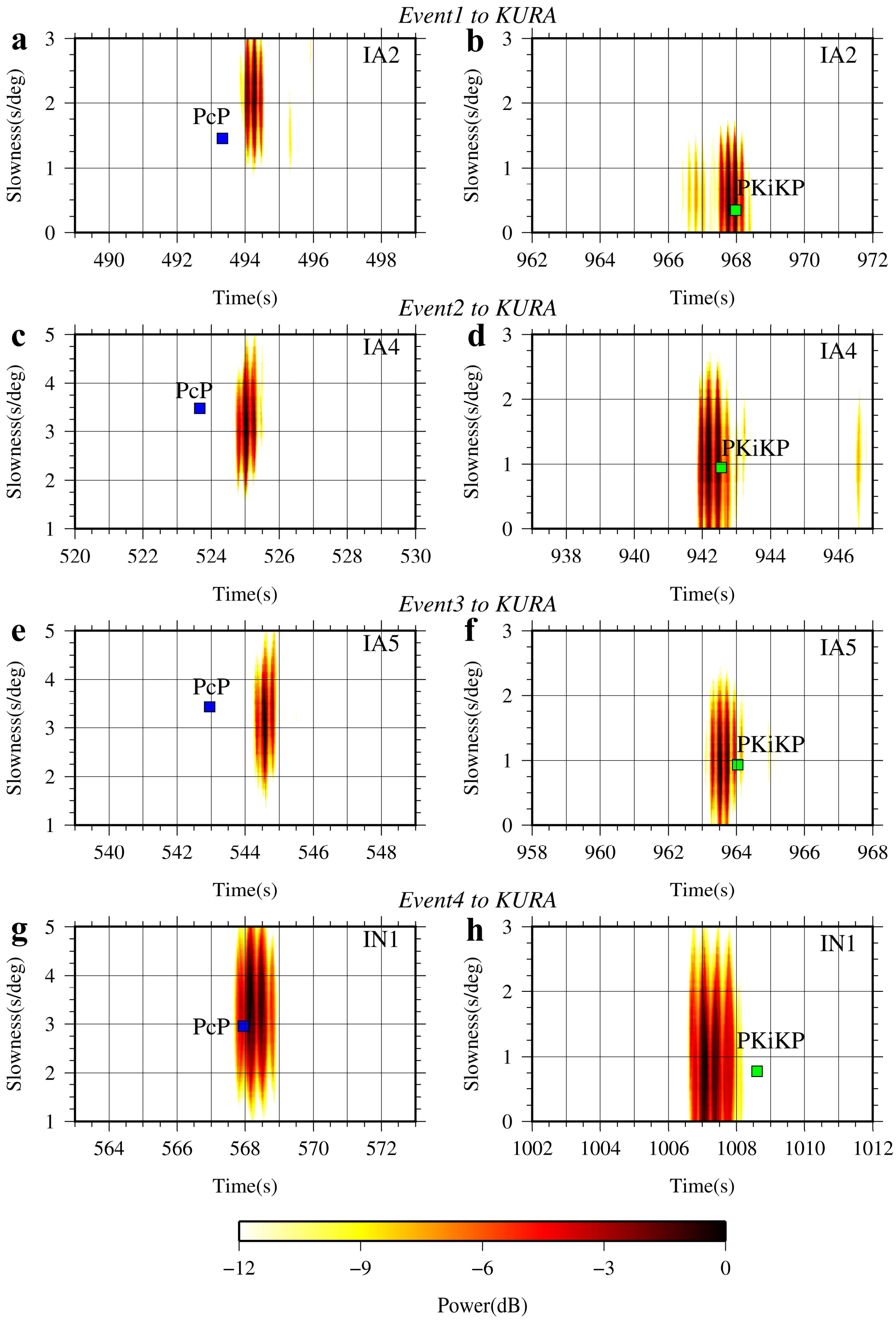

Supplementary Fig. 2. Vespagrams from third-root stacking of PcP and PKiKP observations at KURA for the four events. **a** to **h** PcP stacked results are on the left panel and PKiKP stacks are on the right panel. The predicted slowness and arrival times of the PcP and PKiKP phases from the IASP91 model are marked with blue and green squares, respectively.

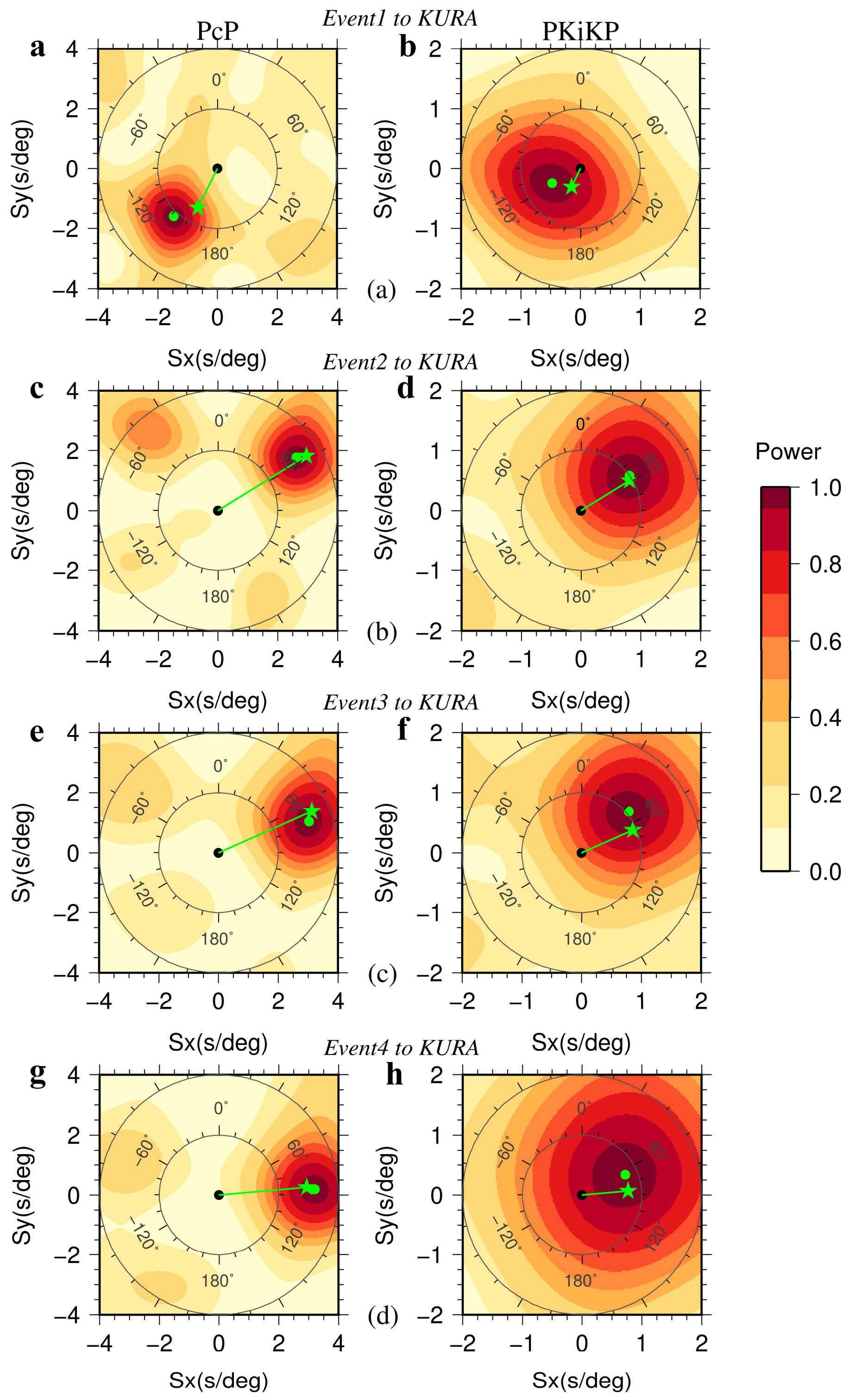

Supplementary Fig. 3. fk analysis of PcP and PKiKP waveforms at KURA for the four events. **a** to **h** The PcP results are on the left panel and PKiKP results are on the right panel. The predicted slowness and backazimuth of the PcP and PKiKP phases marked by green stars. The green circles show the maximum coherence in the fk analysis.

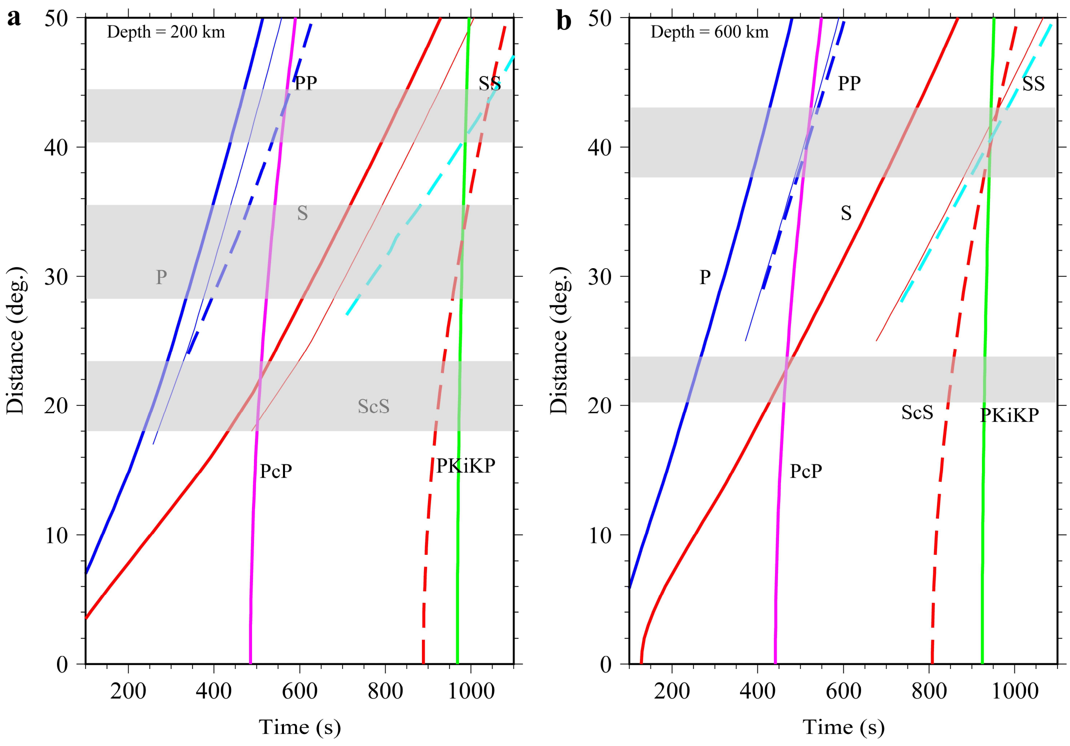

Supplementary Fig. 4. Travel-time curves for P (blue), S (red), PcP (deep pink), PKiKP (green), and other potentially interfering seismic phases. (a) Source depth of 200 km. (b) Source depth of 600 km. Gray shadow zones denote the PcP and PKiKP observations that are ignored to avoid contamination from interfering seismic phases.

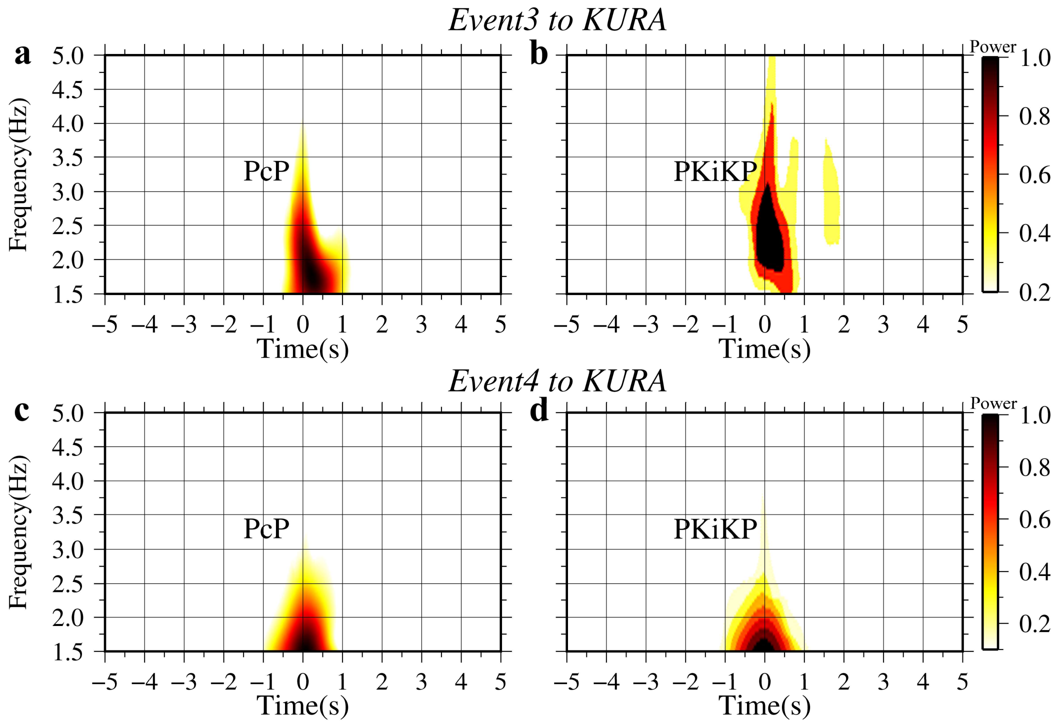

Supplementary Fig. 5. Spectrograms of stack PcP and PKiKP at seismic array KURA form Events 3 and 4. The PcP results are on the left panel and PKiKP results are on the right panel. **a-b** spectrograms of stack PcP and PKiKP at KURA for Event 3. **c-d** Spectrogram results for Event 4. The time zero is the PcP and PKiKP arrival time.

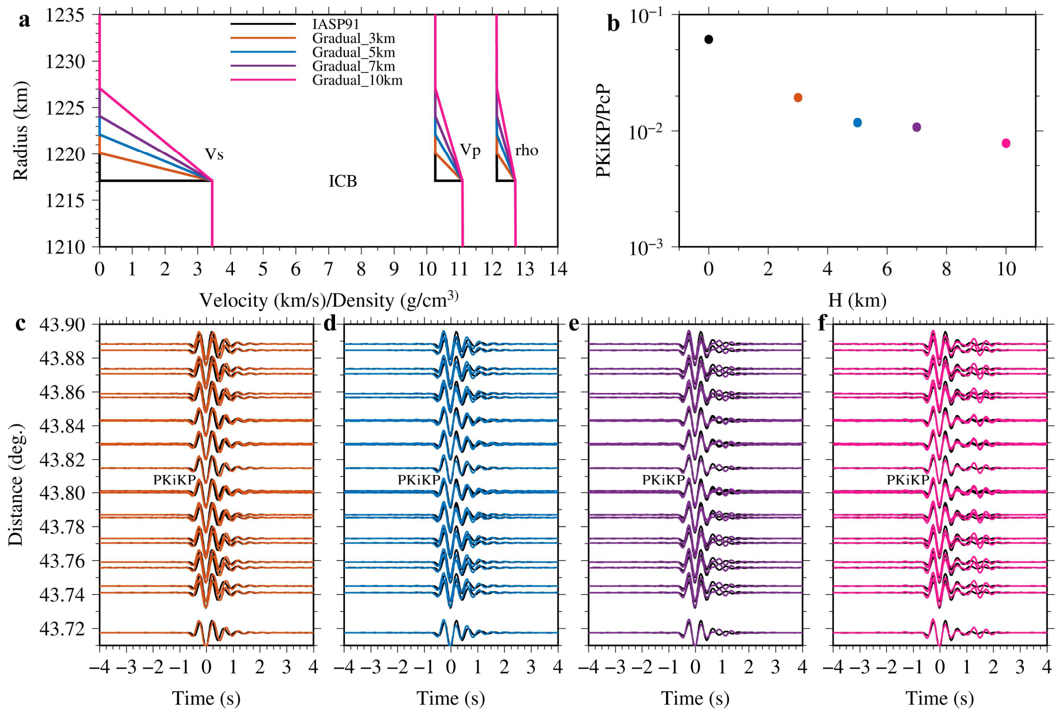

Supplementary Fig. 6. Simulation of effects from gradual transition zones above ICB on PKiKP waveforms. **a** Gradual transition zone models. The thickness of four gradual transition zone models is 3km (brown), 5km (blue), 7km (purple), 10km (deep pink), respectively. **b** The ratios of PKiKP to PcP vs the thickness of gradual transition zones. **c-f** Comparing PKiKP synthetics for the IASP91 model (black) and gradual transition zone models with different thicknesses.

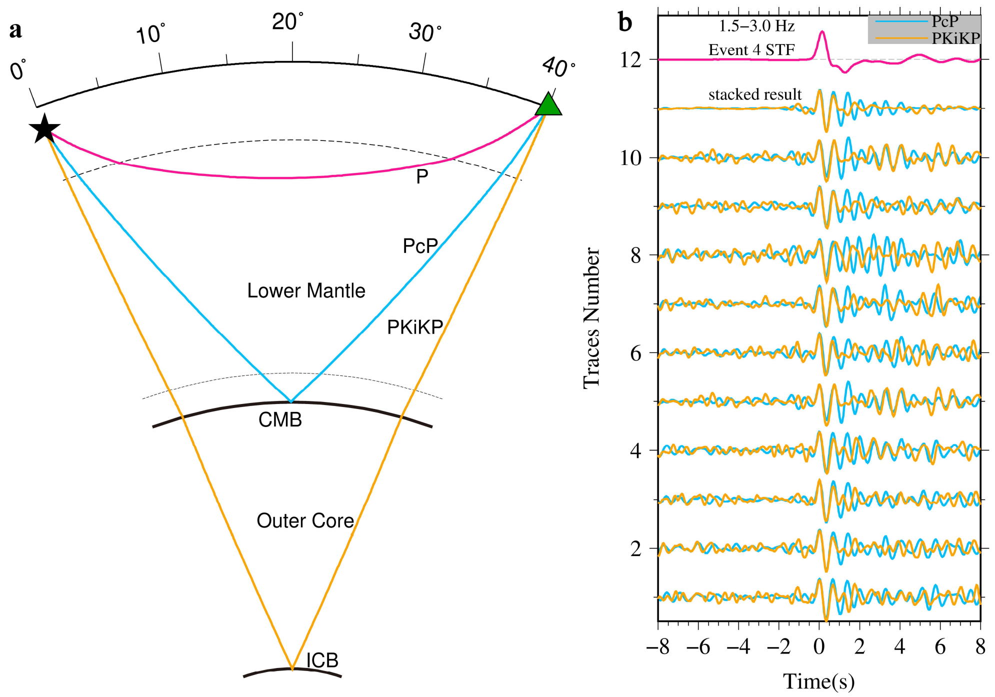

Supplementary Fig. 7. (a) Seismic raypaths of P, PcP and PKiKP phases. (b) Comparisons of the observed PKiKP (orange) and PcP (blue) waveforms at Makanchi array in Kazakhstan for Event 4 with its STF (deep pink) derived from the P waves.

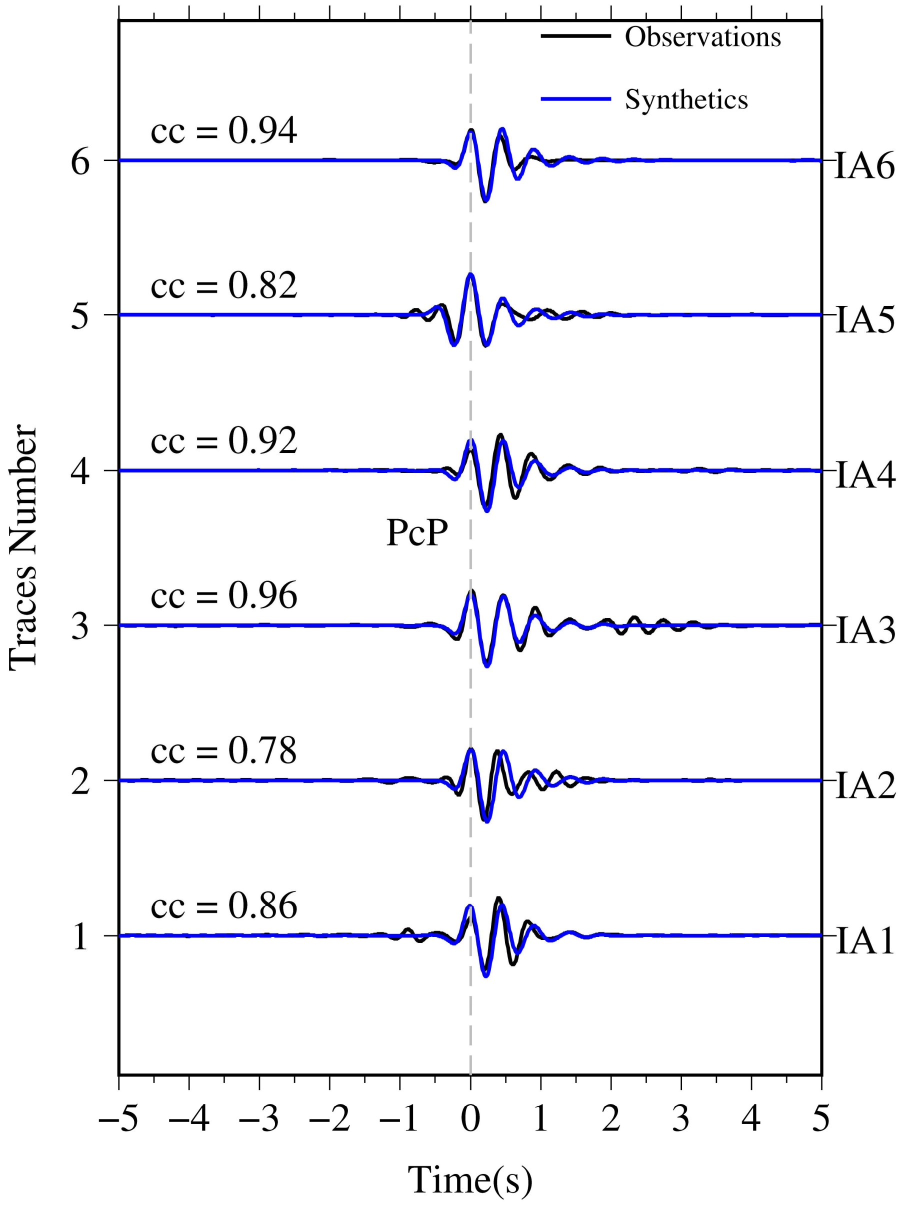

Supplementary Fig. 8. Comparisons of synthetic seismograms (blue traces) with the IASP91 model and stacked observed PcP waveforms (black traces) in Fig. 3b.

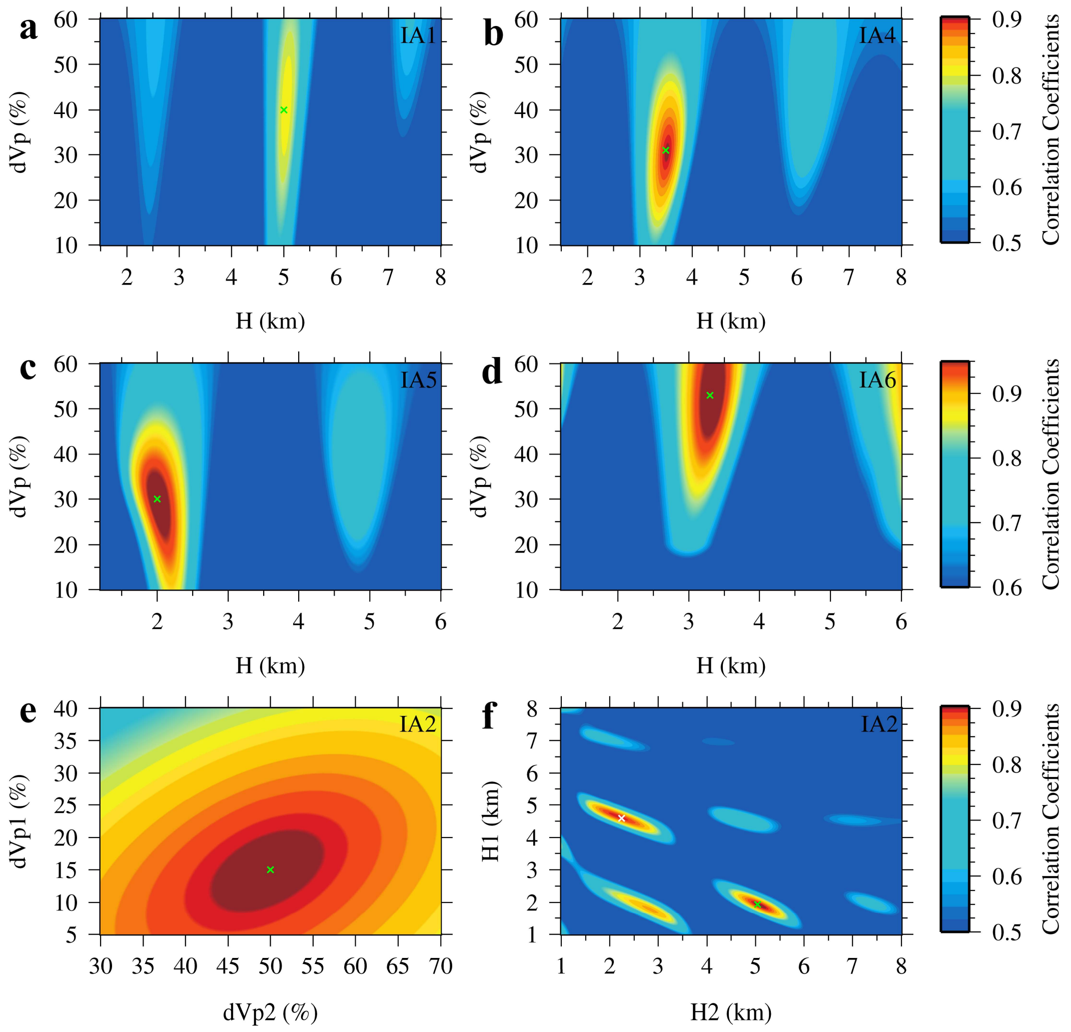

Supplementary Fig. 9. Grid-searching for the optimal model parameters for anomalous ICB regions (IA) in Fig. 3a. The model parameters include mushy zone thickness (H), velocity and density jump within the mushy zone ($dV = \nabla V_{Mz}/\nabla V_{ICB}$). From the empirical relationship Birch Law, we assume $dVp = dVs = d\rho$. The green crosses indicate the optimal model parameters which were adopted to calculate the synthetic seismograms in Fig. 4b, and the models were shown in Fig. 4a. **a-d** Model parameters of the one-layer mushy zone for IA1, IA4, IA5, and IA6 in Fig. 3a. **e** Velocity and density jumps within the double-layers mushy zone for IA2 and IA3. **f** Thickness of double-layers mushy zone for IA2 and IA3. The green cross in (e) and (f) is referred to as ModelICB3a, and the green cross in (e) and the white cross in (f) is referred to as ModelICB3b, respectively.

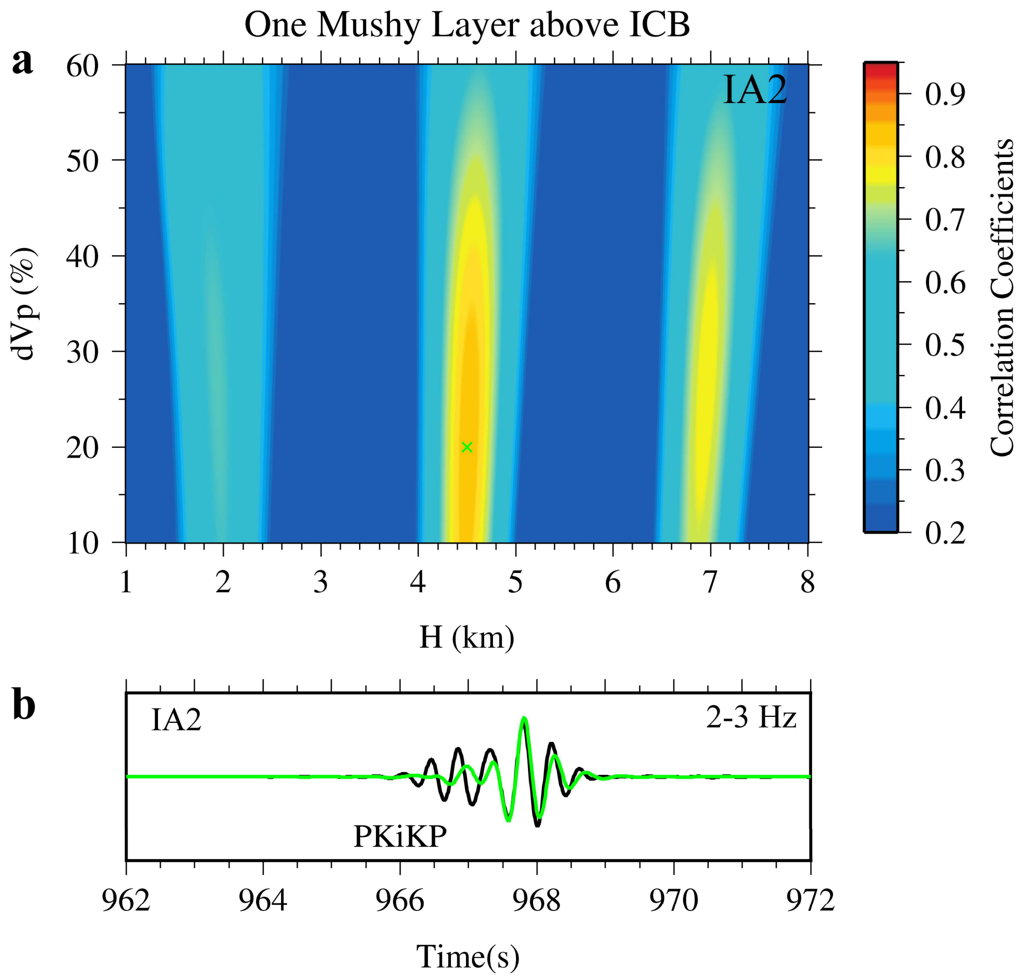

Supplementary Fig. 10. (a) Model parameters of a one-layer mushy zone for IA2 region. (b) Comparisons of synthetic seismograms (green traces) with the best model parameters (the green cross in a) and stacked observed PcP waveforms (black traces) in the IA2 region.

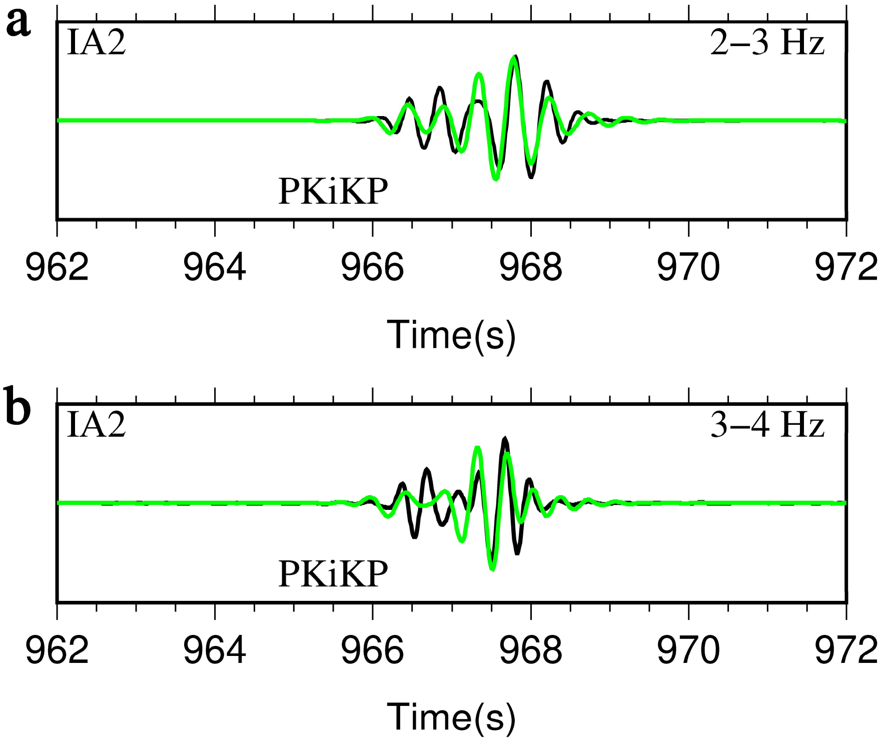

Supplementary Fig. 11. Comparisons of synthetic seismograms (green traces) with the ModelICB3b in Supplementary Fig. 9f and stacked observed PcP waveforms (black traces) of IA2 region in the frequency band of (a) 2.0 to 3.0 Hz, and (b) 3.0 to 4.0 Hz.

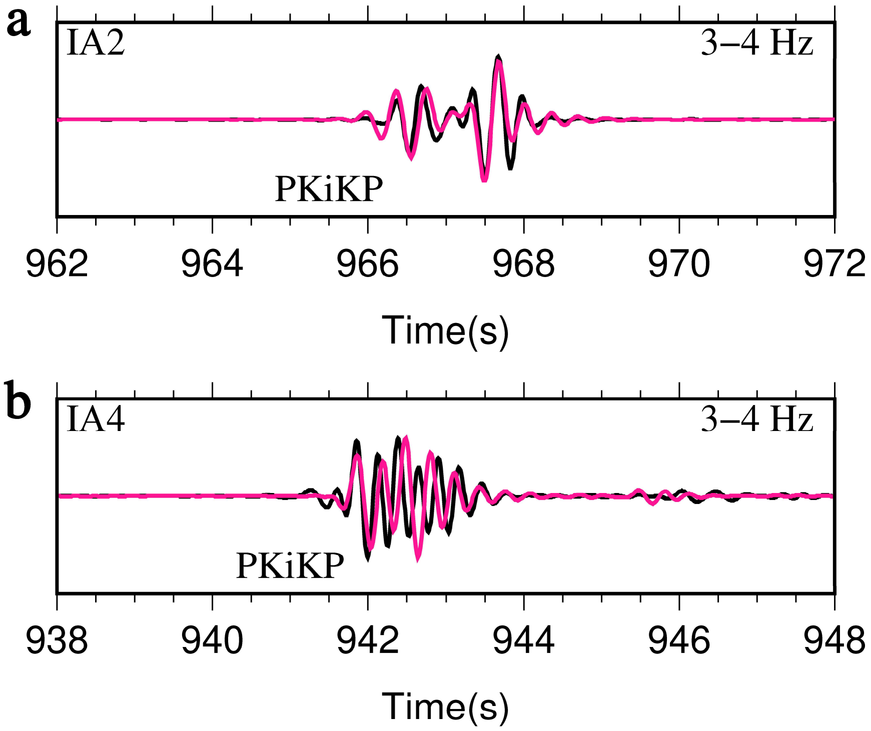

Supplementary Fig. 12. Comparisons of synthetic seismograms (deep pink) with observed PKiKP waveforms (black) in IA2 (**a**) and IA4 (**b**) regions, respectively. The waveforms are band-pass filtered with corners between 3.0 and 4.0 Hz.

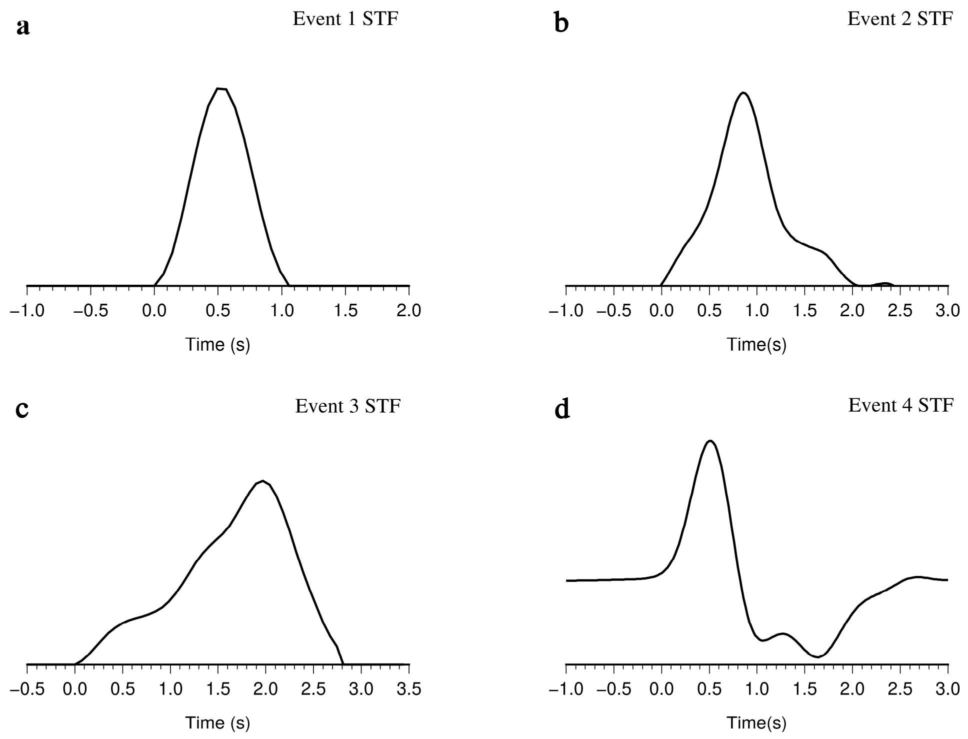

Supplementary Fig. 13. The source time functions (STF) for the four events used in the study. **a** and **c** The STFs of Event 1 and Events 3 were downloaded from SCARDEC source time functions database (http://scardec.projects.sismo.ipgp.fr/), which provide broadband STFs with the SCARDEC method (Vallee & Douet, 2016). We stacked the broadband displacement seismograms of direct P-waves in the distance range of 40-60 degrees to obtain empirical STFs of Event 2 (**b**) and Events 4 (**d**).

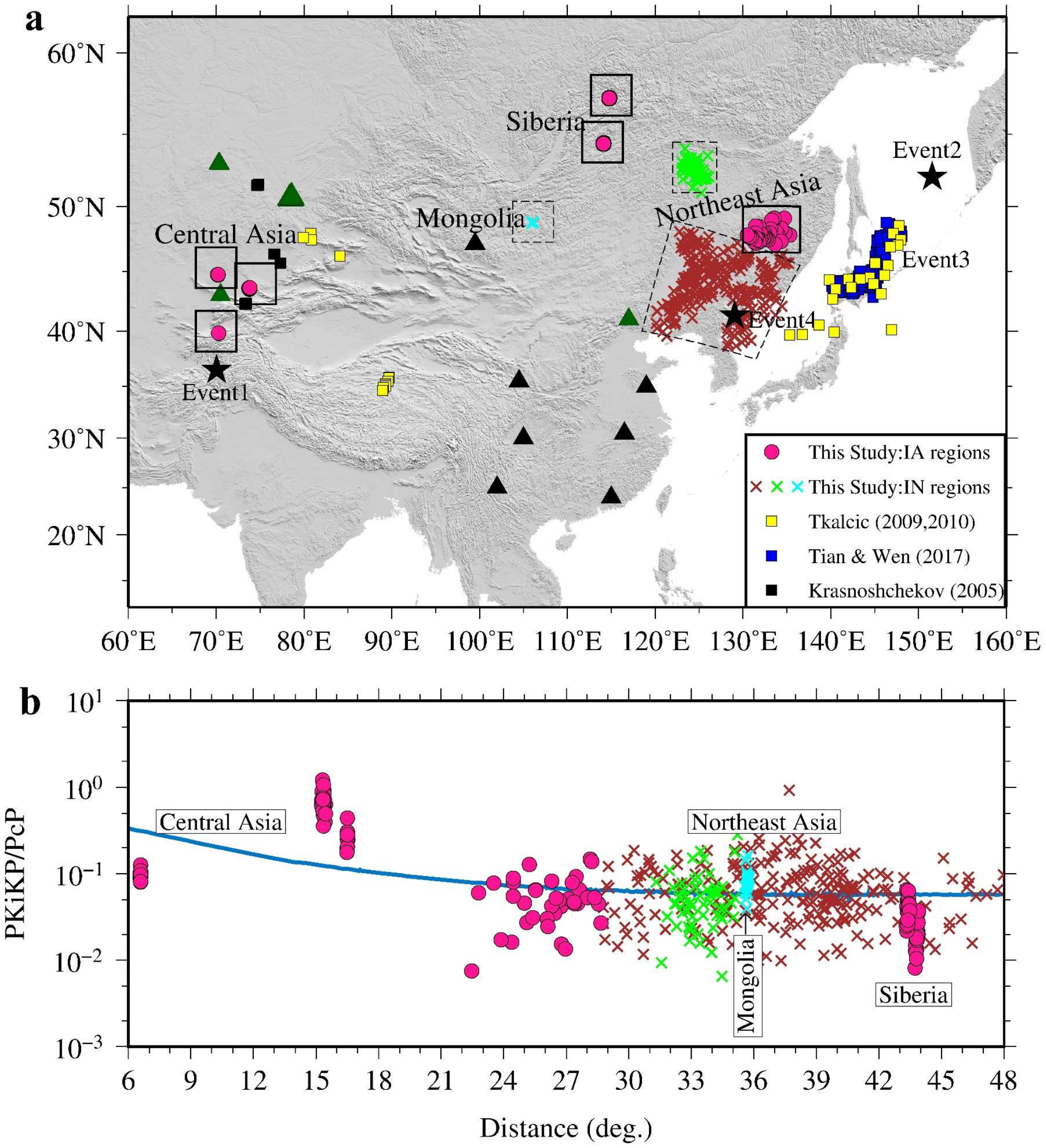

Supplementary Fig. 14. a ICB reflection points distribution in various studies beneath the Central and East Asia. **b** Measured PKiKP/PcP amplitude ratios from observations and synthetics in frequency band of 2-3 Hz. Colored crosses denote the normal ICB regions. Whereas red circles denote the ICB abnormal regions in this study. Squares denote the regions where anomalous amplitudes or waveforms of PKiKP have been observed in previous studies. Blue line is the amplitude ratio of PKiKP to PcP that obtained from synthetic seismograms for the IASP91 model.

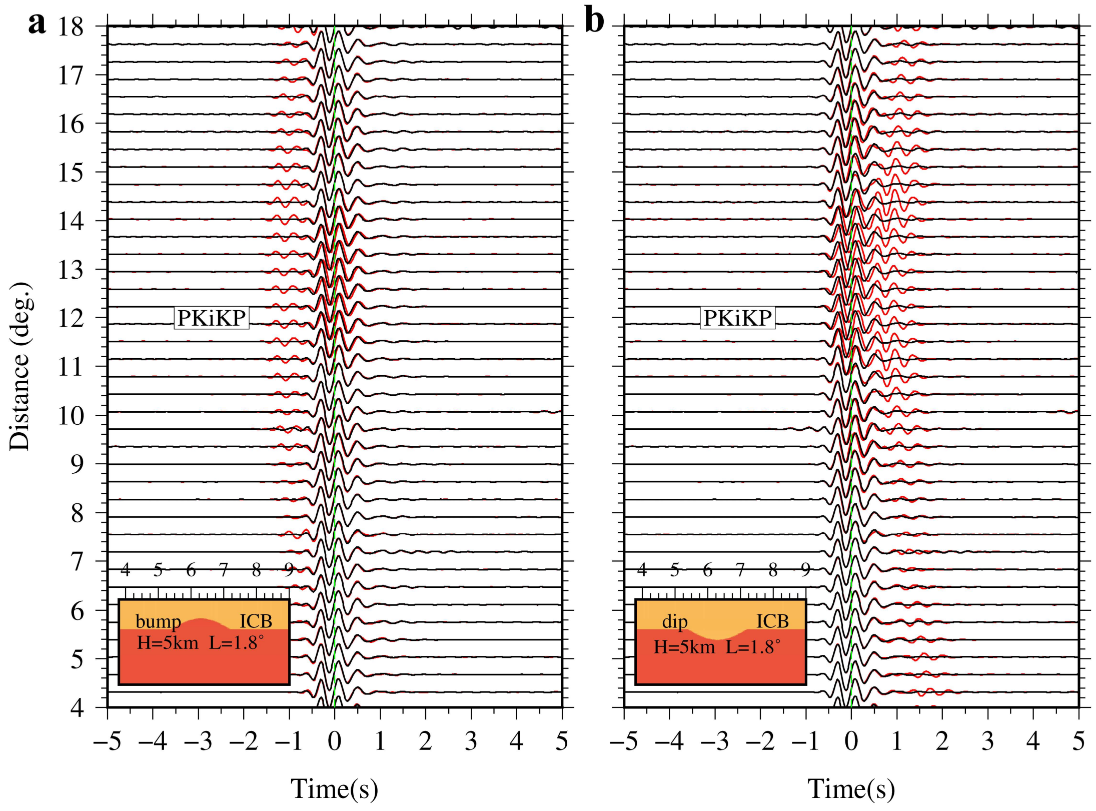

Supplementary Fig. 15. Simulation of effects of the ICB topography on PKiKP waveforms. **a** Sinusoidal bump ICB topography model and its synthetic seismograms (red traces). **b** Sinusoidal dip ICB topography model and its synthetic seismograms (red traces). Black traces are the synthetic seismograms calculated with the IASP91 model.

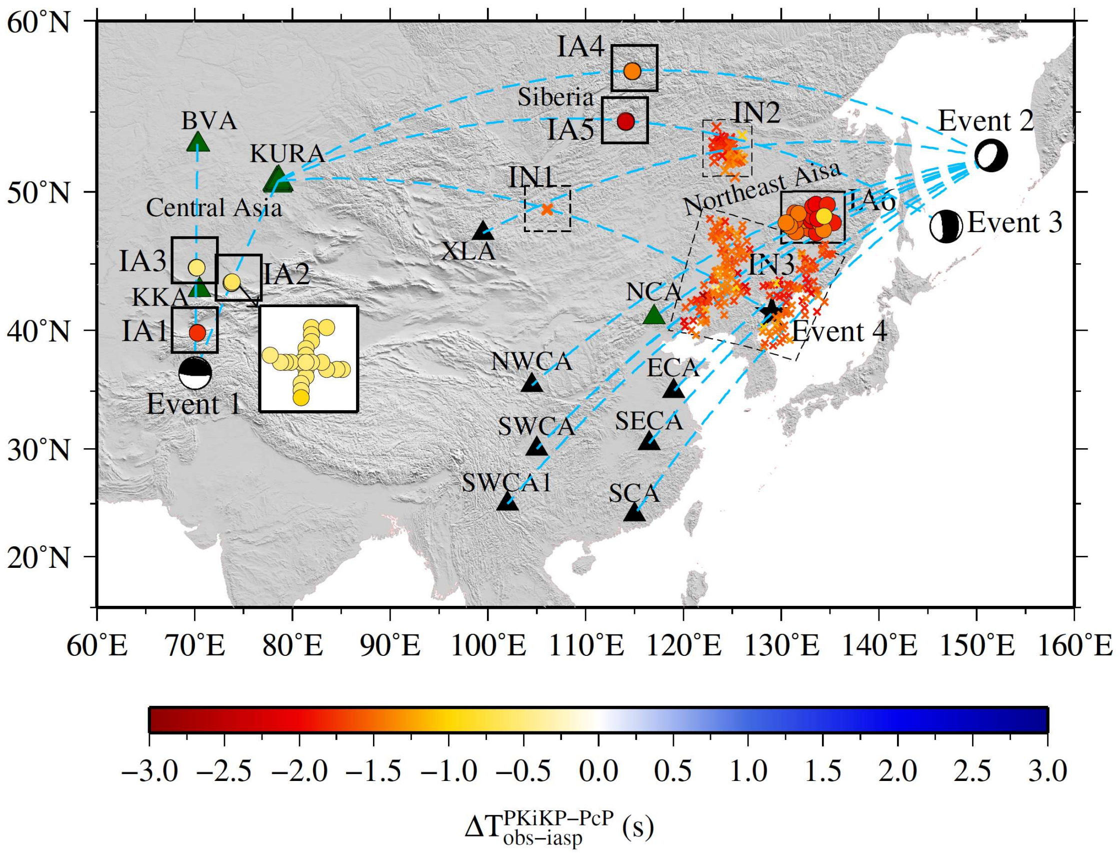

Supplementary Fig. 16. Geographical distribution of ICB reflection points and PKiKP-PcP differential traveltime residuals. Colored crosses denote the reflected points where the stacked PKiKP waveforms are similar to the corresponding PcP. Whereas colored circles denote the reflected points where the stacked PKiKP and the corresponding PcP waveforms show significant differences.

References:

1. Vallée, M. & Douet, V. A new database of source time functions (STFs) extracted from the SCARDEC method. *Phys. Earth Planet. Inter.* 257, 149–157 (2016).
